# Seeing amyotrophic lateral sclerosis in a multi-omic perspective

**DOI:** 10.4103/NRR.NRR-D-25-01010

**Published:** 2025-12-30

**Authors:** Natalie Dikwella, Paul Lingor, Laura Tzeplaeff

**Affiliations:** Department of Neurology, School of Medicine and Health, TUM Klinikum Rechts der Isar, Munich, Germany; German Center for Neurodegenerative Diseases (DZNE), München, Germany; Munich Cluster for Systems Neurology (SyNergy), Munich, Germany (Lingor P)

Amyotrophic lateral sclerosis (ALS) is a rapidly progressing neurodegenerative disease, leading to muscle weakness, paralysis and ultimately death due to respiratory failure. Currently licensed drugs have only very limited effects on slowing down disease progression or biomarkers. Despite numerous successful preclinical analyses, most new drugs fail when translated to clinical trials (Petrov et al., 2017). This is believed to be, in part, due to the multilayer heterogeneity of ALS (e.g., clinical, genetic, and molecular; Tzeplaeff et al., 2024). Studies integrating multi-omic data are still limited, making it difficult to fully understand the biological complexity that characterizes the disease.

**Figure 1:**
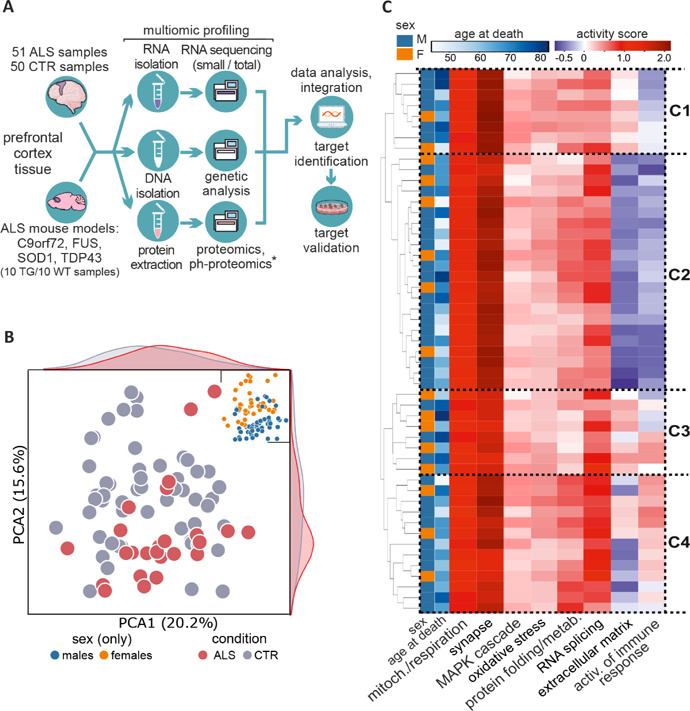
Transcriptomic subcluster and sex-differences in the human post mortem brain. (A) Overview of the sample processing workflow; (B) PCA annotated for disease condition and sex on the 500 most variable genes; (C) hierarchical clustering analysis of ALS-associated pathways reveals four distinct molecular clusters (C1–C4) in ALS patients. Immune response is the primary dichotomizing factor, along with synaptic and RNA functions, separating C1–C2 from C3–C4. The next level of subdivision included extracellular matrix organization, further distinguishing C1 from C2 and C3 from C4. Adapted from Caldi Gomes et al. (2024). ALS: Amyotrophic lateral sclerosis; CTR: control; F: female; FUS: fused in sarcoma; M: male; MAPK: mitogen-activated protein kinase; PCA: principal component analysis; SOD1: superoxide dismutase 1; TDP43: TAR DNA-binding protein 43; TG: transgenic; WT: wild‐type.

Recent work demonstrates the importance of multi-omic integration in understanding ALS pathophysiology and its heterogeneity. First, we investigated the influence of sex in the disease mechanisms; second, we identified molecular subgroups for patient stratification and evaluated their translational relevance in four different transgenic ALS mouse models. Finally, we uncovered molecular determinants involved in ALS and validated one candidate target *in vivo* and *in vitro* (Caldi Gomes et al., 2024; Hausmann et al., 2024). To do so, we conducted a multi-omic profiling of human postmortem tissue from the frontal cortex (region Brodman Area 6) of sporadic ALS patients (*n* = 51) and age-adjusted control subjects (*n* = 50). As the frontal cortex reflects intermediate TAR DNA-binding protein 43 (TDP-43) pathology at the time of death, we postulate that it may reflect molecular changes happening at an early disease stage. Additionally, we compared these results with transgenic mouse models carrying mutations in the four most frequent ALS causing genes (superoxide dismutase 1 [*SOD1*], *C9orf72*, *TDP-43*, and fused in sarcoma [*FUS*]) (**Figure 1A**).

Across transcriptomics, small RNA (mature microRNA and microRNA hairpin) and proteomic we observed a significant impact of sex on human sample repartition (**Figure 1B**). We observed a more pronounced disease signal in males with 73 differentially expressed genes identified in males compared to only 2 in females, 17 differentially mature microRNA in males compared to 9 in females, 99 differentially expressed microRNA hairpin in males compared to 24 in females, and 379 differentially abundant proteins in males compared to 251 in females. Interestingly, while the immune response and extracellular matrix was observed as common dysregulated pathways in both males and females ALS patients, some pathways were altered depending on sex. In the transcriptomic dataset, males showed enrichment in pathways related to synaptic function, while in females, ribosomal functions were enriched. In the proteomic dataset, pathways associated with the development and the extracellular signal-regulated kinase 1/2 (ERK1/2) related pathway were observed in females but not in males. In conclusion, significant sex specific differences have been identified in each omic layer. These findings are consistent with previous evidence that identified sex-specific differences in the blood of patients with ALS (Santiago et al., 2021). Performing preclinical and clinical analyses separating males and females are therefore essential to uncover sex specific differences in biological mechanisms and therapeutics.

Based on the enrichment analysis of the transcriptomic dataset, hierarchical clustering was conducted and revealed four main ALS molecular clusters (C1–C4). Regulation of the immune response emerged as the primary dichotomizing factor along with synaptic and RNA functions, separating C1 and C2 ALS patients from C3 and C4. The next level of subdivision mainly included extracellular matrix (ECM) organization further distinguishing C1 from C2 and C3 from C4 (**Figure 1C**). Weighted gene co-expression analysis identified specific gene networks associated with mitochondrial respiration and synaptic function in C1 and C2, and immune response and RNA splicing in C3 and C4. A similar transcriptomic study in postmortem ALS frontal and motor cortex have identified three molecular clusters: oxidative dysregulation, transcriptional dysregulation, and glial activation (Tam et al., 2019). Using a comparable approach, Marriott et al. (2023) reported three predominant transcriptomic subtypes across two independent postmortem motor cortex cohorts, characterized by neuronal signaling, excitotoxicity, and inflammation. Together with our study, these highlight the central role of immune-related and neuronal/synaptic pathways in distinguishing ALS subtypes. Notably, similar subtypes have also been detected in blood samples from ALS patients, indicating that these molecular signatures can be captured in living individuals and may hold potential for clinical application (Marriott et al., 2023).

Pearson correlation analyses identified some similarities between the C1–C4 ALS clusters and the four transgenic mouse models associated with SOD1, C9orf72, TDP-43, and FUS mutation. SOD1 transgenic mice recapitulated some of the mitochondrial and oxidative stress alterations observed in the C1–C2 ALS subtype, along with ERK1/2 cascade dysregulation. C9orf72 transgenic mice recapitulated some of the immune response alterations observed in the C3 ALS subtype. TDP-43 transgenic mouse model recapitulated some of the mRNA metabolism alteration observed in the C3 ALS subtype. Although the FUS transgenic mouse model lacked robust pathway enrichment, Pearson analyses indicated that they more closely resemble the C3–C4 ALS subtype. This makes sense regarding the similarity between the FUS and TDP-43 protein functions in RNA metabolism. Our study identified ALS molecular subtypes and their associated dysregulated pathways potentially relevant for future drug development and patient stratification in clinical trials. By comparing human molecular subtypes with ALS mouse models, the study offers a novel insight about the translational potential and limitations of each of these models.

Our multi-omic data set from humans and mice demonstrated the potential to identify signaling pathways that could represent potential therapeutic targets for ALS. Although multiple molecular pathways identified in our analysis merit therapeutic validation, we focused on the mitogen-activated protein kinase (MAPK)/ERK pathway. This pathway was altered consistently across several of our data sets and is linked to several ALS-associated defects (Sahana and Zhang, 2021). Alteration of the ERK1/2 signaling was identified in the human proteomic analyses, in the transcriptome of the SOD1 transgenic mouse model, across different WGCNA modules of human and the different mouse models and in the human differential alternative splicing analyses. Key MAPK/ERK pathway members (AKT1, BCL2, MAPK1, and MAP3K1) were central targets of one miRNA of interest (miR-451a) in the human and mice small RNAomic. Integration of the transcriptomic, proteomic, and small RNA data with the Multi-Omics Factor Analysis identified MAPK1, and MAP2K2/MEK2 protein as important contributors in female ALS disease. To note, Multi-Omics Factor Analysis also identified the well-known neurofilament heavy chain ALS marker as an important contributor.

To validate the involvement of the MAPK/ERK pathway in ALS, we used trametinib, a U.S. Food and Drug Administration approved inhibitor of the MAPK/ERK pathway, in both *in vivo* and *in vitro* ALS models. Primary cortical neurons treated with glutamate to induce excitotoxicity showed a significant reduction of cleaved caspase 3 positive cells and an increase in neurite outgrowth upon treatment with trametinib at 20 and 200 nM concentrations. Moreover, the drug was shown to suppress glutamate-induced phosphorylation of ERK1/2. *In vivo*, SOD1-G93A transgenic mice were treated with trametinib (3 mg/kg twice per week) from 9 weeks of age (pre-symptomatic). At 16 weeks, females demonstrated a reduction of several disease markers in the spinal cord, notably a reduction of phosphorylated ERK1/2, p62 aggregates, detergent-insoluble ubiquitin, and SOD1 accumulation. Additionally, trametinib lowered plasma neurofilament light chain at 16 weeks in a first cohort, and in a second independent cohort at 16 weeks and 19 weeks. Together with the reduction of acetylcholine receptor γ subunit levels, this indicates reduced neurodegeneration and muscle denervation in female-treated mice. Trametinib treatment significantly delayed disease onset in females by 5 days, age of paralysis and increased survival by over 6 days. Inhibition of the MAPK/ERK pathway via trametinib seems to have a sex specific impact, as these changes were not observed in males. Alternatively, as phosphorylation of the MEK2 protein is already observed at 9 weeks in male but not in females, it could suggest a greater efficacy of trametinib when administered during the early disease phase. Interestingly, the study of Yue et al. (2023) demonstrated that TDP-43 flies treated with Trametinib exhibited reduced motor deficits and prolonged lifespan. A Phase 1/2 clinical trial investigating the effect of Trametinib treatment in ALS has been performed, but no results are publicly available (NCT04326283). Our data argue for an independent evaluation of male and female patients. Taken together, our study suggests that the MAPK signaling pathway is playing a valuable role in ALS pathogenesis thus justifying further exploration and understanding of this pathway as a therapeutic target using trametinib or other similar drug candidates.

In our study, we demonstrated the potential of clustering ALS patients based on post mortem brain gene expression. However, for evident reasons, brain sampling cannot be implemented in the clinical routine. In the future, novel approaches, analyzing and comparing different circulating biofluids, may provide the most comprehensive insights into ALS biomarkers, helping to identify those most relevant for effective disease stratification. While blood biomarkers are more accessible, less invasive, and cost-effective, cerebrospinal fluid biomarkers offer high specificity and remain essential for gaining deeper insights into pathology in the central nervous system. Alternatively, tears produced by the lacrimal glands, which are directly innervated by the brainstem, could provide direct access to proteins in the central nervous system while remaining easily accessible, non-invasive, and cost-effective. For feasibility in clinical practice, we estimate that the number of biomarkers selected for disease stratification should be sufficient to ensure accurate separation between subgroups, while remaining as low as possible to facilitate routine analyses and minimize costs. We suspect that such stratification strategy could open avenues for more efficient clinical trials by enabling the selection of efficacy-expected subpopulations.

While trametinib appears as one promising drug candidate in our study, we believe that a single-drug therapy targeting a unique pathway may not be enough to effectively treat ALS. To address the heterogeneity of the disease, the validation of a combination of drugs targeting different disease pathways could be another promising strategy. Zebrafish models of ALS can be valuable for efficient and cost-effective screening of drug combinations, allowing for rapid molecular and phenotypic characterization of treatment. Further investigations in more complex systems, such as ALS mouse models and primary cultures, are essential to elucidate the molecular pathways and efficacy of drugs. Notably, matching mouse models with human molecular subtypes for future drug discovery may improve the prediction of treatment outcomes in specific patient subgroups prior to clinical trials. As several gaps between translation from animal models and humans remain, validation of drugs in human induced pluripotent stem cells from ALS patients appears as a new standard to investigate the impact of a treatment in a human context and to develop a more personalized medicine able to tackle disease heterogeneity. Such approaches should enhance the translational accuracy and would allow a safer transition from preclinical to clinical trials.
